# Resistance Pattern and Molecular Characterization of Enterotoxigenic *Escherichia coli* (ETEC) Strains Isolated in Bangladesh

**DOI:** 10.1371/journal.pone.0157415

**Published:** 2016-07-18

**Authors:** Yasmin A. Begum, K. A. Talukder, Ishrat J. Azmi, Mohammad Shahnaij, A. Sheikh, Salma Sharmin, A.-M. Svennerholm, Firdausi Qadri

**Affiliations:** 1 International Centre for Diarrhoeal Disease Research, Bangladesh, Dhaka, Bangladesh; 2 Molecular Microbiology and Microbial pathogenesis program, Division of Biology and Biomedical Sciences, Washington University in St. Louis, MO, United States of America; 3 Department of Microbiology and Immunology, the Sahlgrenska Academy at University of Gothenborg, Sweden; University of Hyderabad, INDIA

## Abstract

**Background:**

Enterotoxigenic *Escherichia coli* (ETEC) is a common cause of bacterial infection leading to acute watery diarrhea in infants and young children as well as in travellers to ETEC endemic countries. Ciprofloxacin is a broad-spectrum antimicrobial agent nowadays used for the treatment of diarrhea. This study aimed to characterize ciprofloxacin resistant ETEC strains isolated from diarrheal patients in Bangladesh.

**Methods:**

A total of 8580 stool specimens from diarrheal patients attending the icddr,b Dhaka hospital was screened for ETEC between 2005 and 2009. PCR and Ganglioside GM1- Enzyme Linked Immuno sorbent Assay (ELISA) was used for detection of Heat labile (LT) and Heat stable (ST) toxins of ETEC. Antimicrobial susceptibilities for commonly used antibiotics and the minimum inhibitory concentration (MIC) of nalidixic acid, ciprofloxacin and azithromycin were examined. DNA sequencing of representative ciprofloxacin resistant strains was performed to analyze mutations of the quinolone resistance-determining region of *gyr*A, *gyr*B, *par*C and *par*E. PCR was used for the detection of qnr, a plasmid mediated ciprofloxacin resistance gene. Clonal variations among ciprofloxacin resistant (Cip^R^) and ciprofloxacin susceptible (Cip^S^) strains were determined by Pulsed-field gel electrophoresis (PFGE).

**Results:**

Among 1067 (12%) ETEC isolates identified, 42% produced LT/ST, 28% ST and 30% LT alone. Forty nine percent (n = 523) of the ETEC strains expressed one or more of the 13 tested colonization factors (CFs) as determined by dot blot immunoassay. Antibiotic resistance of the ETEC strains was observed as follows: ampicillin 66%, azithromycin 27%, ciprofloxacin 27%, ceftriazone 13%, cotrimaxazole 46%, doxycycline 44%, erythromycin 96%, nalidixic acid 83%, norfloxacin 27%, streptomycin 48% and tetracycline 42%. Resistance to ciprofloxacin increased from 13% in 2005 to 34% in 2009. None of the strains was resistant to mecillinam. The MIC of the nalidixic acid and ciprofloxacin of representative Cip^R^ strains were 256 μg/ml and 32μg/ml respectively. A single mutation (Ser^83^-Leu) in *gyr*A was observed in the nalidixic acid resistant ETEC strains. In contrast, double mutation in *gyr*A (Ser^83^-Leu, Asp^87^-Asn) and a single mutation in *par*C (Glu^84^-Ly) were found in ciprofloxacin resistant strains. Mutation of *gyr*B was not found in either the nalidixic acid or ciprofloxacin resistant strains. None of the ciprofloxacin resistant strains was found to be positive for the qnr gene. Diverse clones were identified from all ciprofloxacin resistant strains by PFGE analysis in both CF positive and CF negative ETEC strains.

**Conclusion:**

Emergence of ciprofloxacin resistant ETEC strains results in a major challenge in current treatment strategies of ETEC diarrhea.

## Introduction

Enterotoxigenic *E*. *coli* (ETEC) is the most important cause of diarrhea among children under 5 years of age in developing countries and among travellers in ETEC endemic countries [1, 2]. ETEC express one or both of two types of enterotoxins, namely heat stable (ST) and/or heat labile (LT) toxin which are encoded by the same or separate plasmids [3–5], and may produce one or more of several colonization factors (CFs) [6, 7]. In case of human challenge studies where ETEC strains were used [8], ampicillin was the only antibiotic used for the treatment and clearing of bacteria. When ETEC strains were first isolated in Calcutta in 1968, no antimicrobial resistance was reported. Additionally, a completely uniform sensitivity pattern was also observed in ETEC strains isolated from Apache children in Arizona during 1971 [9]. Studying the effects of antimicrobial treatment in children with ETEC disease has been difficult for two reasons. Firstly, childhood diarrhea is caused by ETEC as well as other bacterial and viral agents. Secondly, the clinical presentations are not sufficient to differentiate among the various pathogenic organisms responsible for diarrhea. One retrospective study of ETEC diarrhea in Bangladeshi patients has shown that in adults, only a minimal effect on the severity of diarrhea was evident when tetracycline was used for treatment [10].

Antimicrobial treatment of traveller’s diarrhea, which is caused by ETEC in about 20–50% of cases, has altered over the years due to increasing antimicrobial resistance [11]. The rise in multidrug resistance in organisms, such as ETEC, can be attributed to the widespread and indiscriminate use of chemotherapeutic agents in countries where diarrhea is endemic. Hence, it has become imperative to use newer antimicrobials for treating traveller’s diarrhea. The antibiotics that have been used so far are ciprofloxacin, doxycycline, sulfamethoxazole-trimethoprim, erythromycin, norfloxacin, ofloxacin, azithromycin and rifamycin [12–14]. Fluoroquinolones are powerful broad-spectrum antimicrobial agents for the treatment of a wide variety of community acquired and nosocomial infections [15]. However, resistance to fluoroquinolones has increased markedly since their introduction in the late 1980s [16]. Recently ciprofloxacin resistance Shigella and Salmonella has emerged lately in Bangladesh [17, 18]. In 2001 ciprofloxacin resistant ETEC was isolated from patients in India [19]. A recent study conducted in 2008–2009 demonstrated the presence of ciprofloxacin resistant ETEC in the drinking water of Bangladesh [20]. No studies have yet been carried out on fluoroquinolones resistance mechanism of ETEC in Bangladesh.

Several studies showed that fluoroquinolones mainly target DNA gyrase, a type II DNA topoisomerase, in gram-negative organisms [21]. DNA gyrase is composed of two subunits, namely A (*gyr*A) and B (*gyr*B). Although the subunit A is highly conserved within bacterial species, most of the mutations reported so far in DNA gyrase are within this subunit [21]. Alterations in subunit A have been documented in a variety of species, including *E*. *coli*, *Shigella*, *Salmonella*, and *Campylobacter* [22, 23]. On the contrary, mutations in subunit B, which is associated with quinolone resistance, have been rarely isolated in clinical samples [24]. In addition to targeting DNA gyrase, fluoroquinolones also target topoisomerase IV. Toposiomerase IV is also composed of two subunits, namely parC and parE, which share significant sequence similarity to the subunits A and B of DNA gyrase [25].

The aim of the present study was to compare the antimicrobial resistance pattern, clonal variation, and sequence analysis of Quinolone resistance-determinig regions (QRDR) of *gyr*A, *gyr*B, *par*C and *par*E mutations in ciprofloxacin resistant ETEC strains.

## Material and Methods

### Ethics statement

The International Centre for Diarrhoeal Disease Research, Bangladesh (icddr,b) carries out a Surveillance of diarrheal patients attending the Dhaka Hospital where every fiftieth patient’s stool specimens were screened for enteric pathogens [26]. Informed oral consent is obtained from the caregivers or guardians on behalf of the patients for collecting specimens. The 2% surveillance is approved by the Institutional Review Board (IRB) of icddrb. The patients or the guardians are assured about the non-disclosure of information collected from them, but are also informed about the use of data for analysis and keeping identity of the participants anonymous.

### Collection and culture of clinical specimens

A total number of 8580 stool specimens were collected from patients, both male (59%) and female (41%), with diarrhea identified from 2% systematic surveillance system [27] at the icddr,b hospital at Dhaka between 2005 and 2009. Two percentage systematic surveillance system means every 50th patient irrespective of age, sex, disease severity or socioeconomic status by administering structured questionnaire. The generated data provides valuable information to hospital clinicians in their decision- making processes and enables the detection of emerging pathogens and early identification of outbreaks and their locations as well as antimicrobial susceptibility pattern. All stool samples were inoculated onto MacConkey agar, Taurocholate-tellurite-gelatin agar and *Salmonella–Shigella* agar plates (Difco, Becton Dickinson & Company, Sparks, MD, USA). ETEC, *Vibrio cholerae*, *Shigellae* and *Salmonellae* were isolated and identified by using standard microbiological and biochemical methods [28]. *Shigella* and *Salmonella* species were confirmed using commercial antisera kits (Denka Seiken, Tokyo, Japan). *Vibrio cholerae* were confirmed using a combination of biochemical and serological methods as described previously [29, 30]. For the detection of ETEC, fresh stool specimens were inoculated onto MacConkey agar and the plates were incubated for 18h at 37°C. Six lactose fermenting individual colonies morphologically resembling *E*.*coli* from MacConkey agar plates were tested immediately for the presence of toxins and CFs as described below.

### Detection of toxin types and CFs

The detection of LT and ST was carried out by multiplex PCR and ganglioside GM1 enzyme- linked immunosorbent assays (ELISA) [31, 32]. The colonies tested for toxin production were also cultured on Colonization factor antigen (CFA) agar plates with and without bile salts [7, 33]. Enterotoxin positive *E*. *coli* colonies from CFA agar plates were tested for the expression of CFA/I, CS1, CS2, CS3, CS4 and CS6 and colonies from CFA agar plus bile plates were tested for the expression of CS5, CS7, CS8, CS12, CS14 and CS17 by monoclonal antibody-based dot blot assays [31, 34]. Strains grown on TSA were tested for CS21 only [35].

### Antimicrobial susceptibility

Antimicrobial agents from Oxoid, Basingstoke, United Kingdom were used to determine bacterial susceptibility by using the guidelines of National Committee for Clinical Laboratory Standards [36]. The antibiotic discs used in the study included ampicillin (10 μg), azithromycin (15 μg), ceftriaxone (30 μg), ciprofloxacin (5 μg), doxycycline (30 μg), erythromycin (15 μg), mecillinam (25 μg), nalidixic acid (30 μg), norfloxacin (10 μg), streptomycin (10 μg), sulfomethoxazole-trimethoprim (25 μg) and tetracycline (30 μg). The minimum inhibitory concentrations (MIC) of nalidixic acid, ciprofloxacin, and azithromycin were determined by the E-test (AB Biodisk, Solna, Sweden) according to the NCCL guideline. *E*. *coli* ATCC 25922, susceptible to all antimicrobials was used as a control strain for susceptibility studies. We performed antibiogram of all ETEC strains collected during the study period and randomly selected the representative nalidixic acid and ciprofloxacin resistant strains. These strains were compared with a few representative nalidixic and ciprofloxacin susceptible strains for genotyping.

### Pulsed-field gel electrophoresis (PFGE)

Intact chromosomal DNA of clinical ETEC strains was prepared and digested with the *Xba*I restriction endonuclease (New England Biolabs) according to the PulseNet program descried elsewhere [37]. DNA fragments were separated by pulsed-field gel electrophoresis on a CHEF-MAPPER apparatus (Bio-Rad) under the following conditions: switching time from 2.2 to 54.2 s at 6 V cm^−1^ for 20 h at 14°C. Analysis of the TIFF image was carried out by the BioNumerics software version 4.5 (Applied Maths, Belgium) with average linkages to generate dendrogram at 1.5% tolerance level using the dice coefficient and unweighted-pair group method.

### PCR and sequencing amplification of *gyr*A, *gyr*B, *par*C and *parE*

Chromosomal DNA was prepared and purified by procedures described earlier [38]. Polymerase chain reaction (PCR) for *gyr*A, *gyr*B, *par*C and *par*E were done according to the methods described earlier [39–41].

#### Nucleotide sequencing

GFX PCR DNA and gel band purification kit (Amersham Pharmacia, USA) were used for the purification of *gyr*A, *gyrB*, *par*C *and par*E PCR amplicons. Sequencing of the purified PCR products were performed by using the dideoxynucleotide chain termination method with an ABI PRISM BigDye Terminator Cycle Sequencing Reaction kit (Perkin-Elmer Applied Biosystems, Foster City, CA, USA) on an automated sequencer (ABI PRISM 310) at the icddr,b core sequencing facility.

#### DNA and protein sequence analysis

The chromatogram sequencing files were inspected using Chromas 2.23 (Technelysium, Queensland, Australia), and contiguous sequences were prepared using SeqMan II (DNASTAR, Madison, WI, USA). Nucleotide and protein sequences were analyzed using the National Center for Biotechnology Information (NCBI, National Institutes of Health, Bethesda, MD, USA) BLAST (Basic Local Alignment Search Tool) server on GenBank database, release 138, in order to find similarities with previously known sequences [42] Multiple sequence alignments developed using CLUSTALX 1.81 [43] sequences were manually edited in the Gene Doc version 2.6.002 alignment editor.

## Results

### Isolation of enteric pathogens from stool specimens

A total of 8580 stool specimens were tested from patients at the icddr,b hospital during the period between 2005–2009 for the isolation of *Vibrio cholerae*, ETEC, *Shigella*, and *Salmonella*. Of 8580 specimens, a total of 3194 pathogens were isolated, of them 21% (1784/8580) were *V*. *cholerae* O1, 12% (1067/8580) were ETEC, 3.0% (259/8580) were *Shigella* spp. and rest 1.0% (84/8580) was *Salmonella* spp. Of *V*. *cholerae* O1, 74% were Ogawa and 26% were Inaba serotypes.

### Toxin types and CFs on ETEC

Among the ETEC isolates, 448 (42%) were both LT and ST followed by the strains producing LT alone 315 (30%) and ST only 304 (28%) (Table 1). In the present study 523 (49%) of the ETEC strains expressed one or more CFs; CFA/I, CS5+CS6 and CS7 were the predominant phenotypes during the study period.

**Table 1:** Number of ETEC strains isolated during the study period and their toxin types.

Year	No. of specimens tested	ETEC No. (%)	ETEC toxin Types		
			LT	ST	LT+ST
**2005**	654	91(14)	24 (26)	42 (46)	25 (28)
**2006**	661	82 (12)	27 (33)	21 (26)	34 (41)
**2007**	2211	291 (13)	100 (34)	70 (24)	121 (42)
**2008**	2369	319 (13)	89 (28)	87 (27)	143 (45)
**2009**	2685	284 (11)	75 (26)	84 (30)	125 (44)
**Total (2005–2009)**	8580	1067 (12)	315 (30)	304 (28)	448 (42)

### Antibiotic resistance pattern

The majority of the ETEC strains isolated during the study period showed high resistance (Table 2) to the 12 different antibiotics tested, including members of the quinolone (nalidixic acid) and fluoroquinolone groups (ciprofloxacin or norfloxacin). Antibiotic resistance pattern was as follows: ampicillin 66%, azithromycin 27%, ciprofloxacin 27%, ceftriazone 13%, sulfomethoxazole-trimethoprim 46%, doxycycline 44%, erythromycin 96%, nalidixic acid 83%, norfloxacin 27%, streptomycin 48% and tetracycline 42% respectively. Resistance to ciprofloxacin increased from 13% in 2005 to 34% in 2009. However none of the strains was resistant to mecillinam.

**Table 2:** Resistant pattern of ETEC strains isolated during the study periods (2005–2009).

Name of antibiotics	2005* (N = 91) (%)	2006* (N = 82) (%)	2007* (N = 277) (%)	2008* (N = 191) (%)	2009* (N = 262) (%)
**Ampicillin (A)**	53	60	66	70	71
**Azithromycin (Azm)**	5	13	22	32	33
**Ciprofloxacin (Cip)**	13	26	25	26	34
**Ceftriaxone (Cro)**	4	6	13	13	17
**Doxycline (D)**	33	35	46	49	44
**Erythromycin (E)**	95	95	96	97	100
**Nalidixic Acid (Na)**	70	76	80	90	87
**Norfloxacin (Nor)**	13	26	25	26	34
**Streptomycin (S)**	35	39	51	47	53
**Sulfomethoxazole-trimethoprim (Sxt)**	38	49	49	51	41
**Tetracycline (T)**	31	32	43	49	44

* Number of ETEC strains tested during the year

Most of the fluoroquinolone resistant ETEC strains tested were CF negative 84% (n = 204). All ETEC strains were tested for 13 colonization factors using 13 specific monoclonal antibodies. A probable reason for the above results may be that other colonization factors were not tested. Moreover, ETEC strains might have possibly lost the plasmids harboring the CF genetic elements because of repeated subculture in vitro or a mutation in genetic locus. Multiple antibiotic resistance patterns and the minimum inhibitory concentrations (MIC) of the representative ciprofloxacin resistant strains are shown in (Table 3). The range of MIC values of the ciprofloxacin was 32 μg/ml and nalidixic acid 64–256 μg/ml.

**Table 3:** MIC values and amino acids changes in *gyr*A and *Par*C in representative ETEC strains isolated in Bangladesh.

Strain	Toxin types	CFs	Resistance pattern	MIC (μg/ml)			Substitution in QRDR^c^					
				Na	Cip	Azm	*gyr*A				*Par*C	
**2533700**	LT+ST	CS5+CS6	Sensitive	2	0.012	0.38	-	-	-	-	-	-
**2960200**	LT+ST	CS1+CS3+CS21	Sensitive	0.008	1	0.75	-	-	-	-	-	-
**2511850**	ST	CFA/I	E Na Sxt T	256	0.19	1	Ser^83^	Leu	-	-	-	-
**2526600**	LT	Negative	A Cip D E Na Sxt T	256	32	4	Ser^83^	Leu	Asp^87^	Asn	Glu^84^	Ly
**2848150**	LT	Negative	A Azm Cip D E Na Sxt T	256	32	256	Ser^83^	Leu	Asp^87^	Asn	Glu^84^	Ly
**0008200**	ST	Negative	A Azm Cip Cro D E Na S Sxt T	256	32	256	Ser^83^	Leu	Asp^87^	Asn	Glu^84^	Ly

### Identification of mutations and clonal variations

Further investigation using gene-sequencing QRDR (Quinolone resistance-determining region) of *gyr*A, *gyr*B, *par*C and *par*E was performed to investigate the mutations of nalidixic acid and ciprofloxacin resistant ETEC strains. A single mutation (Ser^83^-Leu) in *gyr*A was observed in the nalidixic acid resistant ETEC strains. In contrast, double mutation in *gyr*A (Ser^83^-Leu, Asp^87^-Asn)) and a single mutation in *par*C (Glu^84^-Ly) was found in all of the most recently isolated fluoroquinolone resistant strains in diarrheal patients. Mutation of *gy*rB was not found either in nalidixic acid or ciprofloxacin resistant strains. No mutation in *gyr*A, *gyr*B, *par*C and *par*E was observed by gene sequence analyses among the quinolone and fluoroquinolone susceptible ETEC strains.

**Fig 1:**
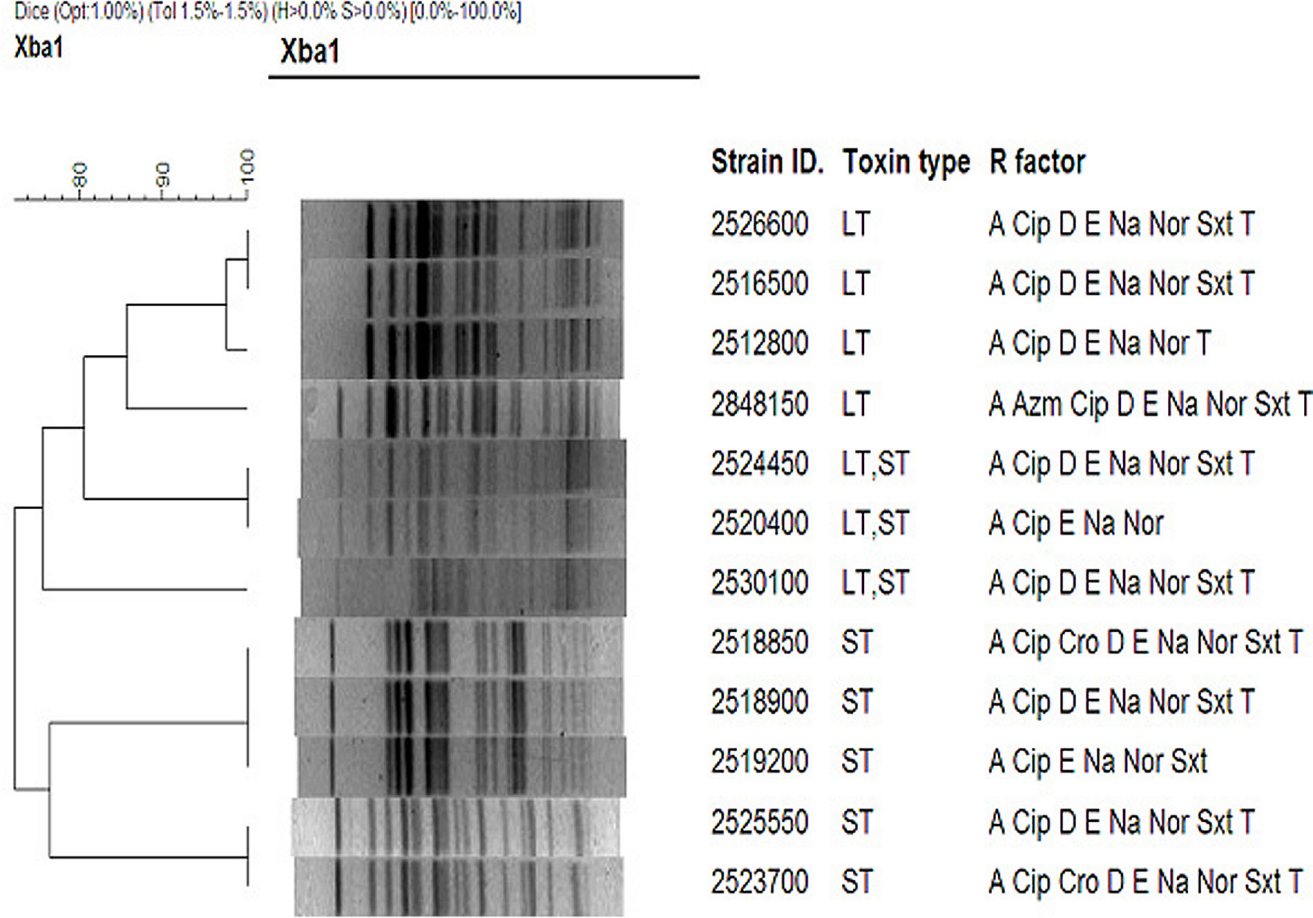
BioNumeric software generated dendrogram. **The distances shown were calculated by the dice similarity index of PFGE *Xba*I profiles for clinical ETEC isolates isolated from Bangladesh.** The scale shows the degree of similarity (%).

PFGE typing of representative ciprofloxacin resistant strains (n = 12) of different ETEC toxin types (LT, LT/ST and ST ETEC) showed that more than one clone was detected within a specific toxin type (Fig 1).

## Discussion

Analyses of data from patients hospitalized in diarrheal hospitals, have shown that patients with ETEC diarrhea are often given antibiotic treatment together with rehydration therapy [44]. The treatment is similar to that given to patients with *V*. *cholerae* induced diarrhea. This is done to reduce duration of hospital stay as well as decrease of transmission of diarrheal pathogens in the environment. However, careful monitoring of antimicrobial sensitivity in treated patients is generally not carried out. Our results show that ETEC is also following a similar trend of increased antibiotic resistance to drugs commonly used for treatment of *V*. *cholerae* O1 and other diarrheal infections [45, 46].

Our present analysis showed that 88% of ETEC strains isolated from stools of diarrheal patients attended at a 2% surveillance system at icddr,b Dhaka Hospital in Bangladesh during five years (2005–2009) were multidrug resistant. Resistance was observed to nalidixic acid and ciprofloxacin as well as reduced sensitivity to azithromycin and ceftriaxone. There have been reports from India and Japan on multidrug resistance to the fluoroquinolones in ETEC strains isolated during outbreaks [19, 46]. In this study no CFs were detected in 84% of the ciprofloxacin resistant strains. Similar finding was also observed in India [19]. A recent study, reported the presence of ciprofloxacin resistant ETEC in environmental samples during 2008–2009 in Bangladesh [20]. This change is trend in Bangladesh is alarming as these antibiotics are used for the treatment of enteric infections.

Quinolones are broad-spectrum antibacterial agents that act by inhibiting the DNA gyrase and topoisomerase IV activities [47]. We showed that the isolated ciprofloxacin resistant ETEC strains on sequencing had a single mutation in *gyr*A gene in the nalidixic acid resistant strains and double mutation in *gyr*A in the ciprofloxacin resistant ETEC strains. In addition, a single mutation in *par*C was found in fluoroquinolone resistant ETEC strains. This suggests that *gyr*A *and par*C are intracellular targets of quinolones and fluoroquinolones, and that mutation in these genes are associated with fluoroquinolones and/or quinolones resistance in *E*. *coli*, which is also supported by previous studies [40, 48]. No mutation in *gyr*B was observed in the ciprofloxacin and nalidixic acid resistant ETEC strains examined in our study. Among the quinolone and fluoroquinolone-susceptible ETEC strains, however, no mutations were observed in *gyr*A, *gyr*B, *par*C and *par*E by sequence analysis. PCR analysis also showed that none of the strains was found to carry the *qnr* gene. The *qnr* gene encodes an immune protein which protects *E*. *coli* gyrase from quinolone inhibition. Hence, along with these factors, additional factors may also be involved in mediating fluoroquinolone resistance in ETEC. Although *in vitro* drug resistance pattern not always be related to *in vivo* activity of the drug, it may indicate the trend of the sensitivity. Our results show that ETEC strains with fluoroquinolone resistance is increasing in Bangladesh and also that the gyrA and parC genes were mutated. There are important informations for therapeutic purposes. However it may mention that in vitro drug resistance pattern has not always been found to correlate with in vivo activity and indicate treatment successor.

Dendogram analysis of representative ETEC strains indicated that Ciprofloxacin resistance in ETEC was not clonally related, since Cip resistance due to *gyr*A gene mutation was acquired by diverse clones of ETEC. Thus, similar to earlier observations, we did not find any relationship with the toxin phenotype, CFs or antibiotic resistance pattern [6] among the ciprofloxacin resistant strains.

In summary, multidrug resistance pattern of ETEC strains especially to fluroquinoles is a matter of great concern for appropriate management of ETEC diarrhea. This also underscores the need for the dissemination of this information not only for control of endemic ETEC diarrheal diseases but also for traveler’s diarrhea. Thus immunoprophylaxis guarantee with effective vaccines is required to prevent ETEC diarrheal cases globally.
